# Large dynamic range stellar radiation simulation optical system

**DOI:** 10.1038/s41598-023-49730-w

**Published:** 2024-01-19

**Authors:** Yu Zhang, Yuegang Fu, Qiang Liu, Li Wang, Songzhou Yang, Shitong Liang, Jian Zhang, Jun Zhong, Bin Zhao, Yao Meng

**Affiliations:** 1 College of Optoelectronic Engineering, Changchun University of Science and Technology, Changchun, 130022 Jilin China; 2 Jilin Province Optoelectronic Measurement and Control Instruments Engineering Research Center, Changchun, 130022 Jilin China; 3 Key Laboratory of Optoelectronic Measurement and Control and Optical Information Transmission Technology, Ministry of Education, Changchun, 130022 Jilin China; 4 Beijing Institute of Control Engineering, Beijing, 100190 China

**Keywords:** Aerospace engineering, Astronomical instrumentation

## Abstract

For the current stellar spectral simulation can not realize the stellar color temperature information with large dynamic range simulation, this paper proposes a broad spectrum high-resolution subdivision and spatial beam zoning modulation combined with a large dynamic range of stellar radiation information simulation method, designed a kind of imaging and non-imaging stellar radiation information simulation optical system, using an optical system to achieve multi-color temperature spectrum and large dynamic range stellar simulation. The experimental results show that the designed system can simultaneously achieve the spectral simulation accuracy (single point evaluation) better than ± 7% in the range of spectral 450–1000 nm and color temperature 3000–11,000 K; on the premise of ensuring the spectral simulation accuracy, the magnitude simulation range reaches 0 to  + 12 Mv, and the magnitude simulation accuracy is better than ± 0.05 Mv; Accurate simulation of stellar spectral information and energy large dynamic range tuning is realized, and the system is extended. The system function has been extended to realize the switching of broadband and narrowband modes, The half-peak width of the narrowband output beam is better than 4.1 nm, which extends the application of the spectral simulation technology and provides the theoretical and technical basis for the ground calibration of the development of the high-precision stellar radiation information ground simulation system.

## Introduction

Stellar radiation simulation technology has important applications in the field of aerospace. It is an important tool for ground testing and radiation calibration of spacecraft, satellites and other attitude control systems, remote sensing instruments, etc.^1,2^. In the meantime, along with the development of emerging navigation modes such as astronomical spectral velocimetry navigation and the increasing demand for the development of miniaturized and lightweight micro-nano-satellite platforms^3–6^, there is a higher demand for space ground calibration equipment, such as the accuracy of the spectral information simulation, the flexible working modes of the wide and narrow-band spectral outputs, and the regulation of the energy in a wide range. At present, the ground calibration equipment has technical problems of mutual constraints, such as high-precision spectral simulation and large dynamic range stars, which makes the simulation of ground stellar radiation information distorted. At the same time, it does not have the capability of high-precision narrow-band spectral simulation, which seriously restricts the accuracy of ground calibration of sensitizers, remote sensing equipment, etc., the calibration cycle, and the validation of new theories and new methods.

In terms of accurate simulation of spectral information, due to the low accuracy of the simulated spectra of the current ground calibration equipment, it cannot simulate the spectral information of the real starpoints observed by the star sensitizers in the universe. Whereas each detector device has its own intrinsic spectral response function, this mismatch between the simulated star point spectral information and the real star point spectral information will bring certain errors to the identification of the star map and the measurement of the star point position of the star sensitizer^2^. In terms of energy regulation over a wide range, the highest detectable magnitude of star sensitizers on satellite platforms at home and abroad is close to + 12 Mv, and the detectable magnitude of large aperture pointing optical telescopes under long exposure time is close to + 16 Mv^7–13^. However, the ground-based calibration equipment is limited by the irradiance measurement equipment and its accurate magnitude simulation range is generally concentrated in 0 to + 6 Mv^14–16^, and higher magnitudes are realized by the attenuation method, which cannot guarantee the accuracy of the simulation of the magnitude after several attenuations. The calibration of the magnitude detection limit of star-sensitive devices, large aperture, and other equipment can only rely on the field stargazing test, which is obtained through the star chart comparison method. Still, the test period of this method is long, and it is affected by the weather and the interference of the surrounding stray light, and the test is not reproducible. In view of the above problems, this paper carries out research on the ground calibration system with large dynamic range and stellar radiation information simulation to solve the problems of lack of accurate simulation of spectra, high magnitude, and small energy range adjustment in the ground calibration test.

The stellar radiation simulation system is essentially a tunable light source, and many studies have been conducted at home and abroad in tunable light source technology. In 2000, Fateley et al.^17^ first proposed the design idea of using spatial light modulation techniques to achieve modulation of the output spectrum of the light source. In 2005, MacKinnon designed a spectrally programmable light engine for biochemistry and biomedical analysis by providing various narrow-band spectral distributions. Because of its application of prismatic beam splitting, the nonlinearity of dispersion makes the beam bandwidth of the infrared part broadened^18^. In 2006, Brown et al.^19^ designed a tunable light source applied to radiometric spectroscopy, and the system used fiber-coupled collimated output and prismatic beam splitting, which has not only dispersion nonlinearity but also low energy utilization of the system. In 2012, Liu et al.^20^ designed an integrating sphere light source with a combination of xenon lamp and light-emitting diode, which has some spectral modulation capability, but its spectral simulation accuracy is low. In 2013, Yuan et al.^21^ designed an integrating sphere reference light source for optical remote sensing radiation calibration with a uniformity of up to 99%, which does not have spectral modulation capability due to the use of a single light source. In 2015, Sun et al.^22^ designed a 4 m diameter uniform extended calibration light source. The uniformity of the emitted beam can reach 98%, and the maximum integrated radiometric luminance is 222.62 W/(m^2^-sr). Still, all use a single light source, without considering the radiation calibration light source on the observation target spectral distribution simulation. In 2015, Liu et al.^23^ designed a multi-color temperature integrating sphere light source based on LED. In 2016, Ding Li innovated a spectrally programmable light source utilizing a Digital Micromirror Device (DMD). This technology dissects wide spectral beams, facilitating broad-band spectrum analysis^24^. In 2018, Wang et al. employed the spatial light modulation feature of DMD to create a double-grating, spectrally tunable light source, structured on the Ebert Fastie model. This innovation yielded narrow-band spectral outputs with monochromatic light uncertainty levels of 4.68% at 450 nm, 1.54% at 550 nm, and 1.48% at 654.6 nm, though the system's architecture is notably complex^25^. 2020, Xu Da designed an Offner-type wide dynamic range radiation calibration light source^16^, which achieves high accuracy simulation of the spectrum and has two operating modes of broadband and narrowband. Still, its dynamic range is only 42.2–0.064 W/m^2^ (i.e., 0 to  + 6 Mv simulation range). At present, the method of expanding the dynamic range of the light source generally applies an external light source to compensate for the dynamic performance of the system^15^. Still, the external light source will cause the system's structure is huge, complex control. The modulation of the external light source is generally variable diaphragm attenuation, variable diaphragm driven by a motor to control the incident beam aperture, due to its low control accuracy, can not achieve a large dynamic range of stellar stars and other simulations, and in the process of energy modulation The accurate simulation of stellar color temperature information cannot be guaranteed.

Through the analysis of the current situation of domestic and foreign research in the spectral accurate simulation, the advantages of spatial light modulation technology are obvious, because the spectral accurate simulation itself requires high modulation capacity, causing its disadvantage is generally low energy, the system energy dynamic range is small; in the energy simulation, the integrating sphere light source has the advantage of volume, can enhance the system energy and dynamic range by adding lamp groups, so it has large energy In terms of energy simulation, the integrating sphere light source has a large energy dynamic modulation range because it has the advantage of size and can enhance the energy and dynamic range of the system by adding lamp groups, so it has a large energy dynamic modulation range. In order to solve the existing stellar spectral simulation technology exists in the small range of energy dynamic adjustment (under the premise of ensuring the accuracy of stellar spectral simulation), this paper proposes a broad-spectrum high-resolution subdivision and spatial light zoning modulation combined with a large dynamic range of stellar radiation simulation optical system design method, share a set of systems to simultaneously achieve the accurate simulation of broadband stellar spectra, the large dynamic range of magnitude adjustment, and the system's operating mode is extended to realize the output of wide spectral range stellar color temperature and narrow band monochromatic beam, which extends the application of spectral simulation technology.

## Composition and working principle of a large dynamic range stellar radiation simulation system

**Figure 1:**
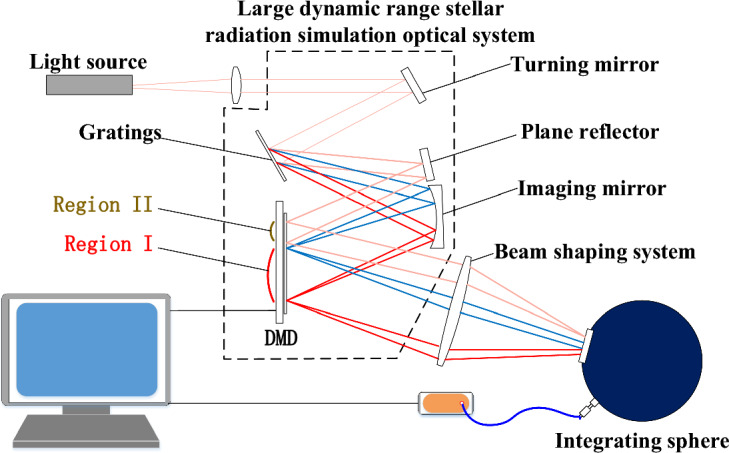
Schematic diagram of the large dynamic range stellar radiation simulation system.

The large dynamic range stellar radiation source is mainly composed of a Light source system, a Large dynamic range radiation spectroscopy system, a digital Micromirror DMD, a beam coupling system, and an integrating sphere, as shown in Fig. 1.

A super continuous laser is selected as the light source for the stellar radiation simulation system. The laser beam is incident into the Large dynamic range radiation spectroscopy system after passing through a collimated beam expansion system. Using a grating, the broad-spectrum beam is precisely beam split to divide the array surface of the DMD into two regions. For -1-level diffraction, the subdivided beams are imaged sequentially in the region I on the array face of the DMD using an imaging mirror. For level-0 diffraction, a planar reflector is used to reflect directly into region II of the DMD. The DMD modulates the beams in different regions. The free modulation of the spectrum in broadband mode and the output of narrowband monochromatic beam are achieved by selecting the Micromirror state in region I. By selecting the micromirror state in Region II, the dynamic amplitude modulation range of the system is expanded.

Among them, the large dynamic range radiation spectroscopy system is the core part of the radiation simulation source. Its main role is to accurately spectroscopy the incident broad spectrum beam and imaging on the DMD array surface located in the best image plane position. The quality of imaging directly affects the optical performance of the stellar radiation simulation system. The spatial light modulator is a surface array device, so the large dynamic range radiation spectroscopy system needs to have good imaging quality, to ensure that the imaging spectrum is flat, with no spectral overlap, spectral displacement and other phenomena. Various color light corresponds precisely to the relevant channel of the spatial light modulator. The spatial light modulator is controlled to realize the modulation of the spectrum in the corresponding channel, and the modulated light beam is homogenized by the light guide element and output.

## Large dynamic radiation spectroscopy optical system design

### Spectral simulation accuracy analysis

The spectral simulation is based on the independence of light and the principle of spectral superposition^26^, so the simulation of the spectrum can be expressed by Eq. (1).

1 $$ L\left( \lambda \right) = \sum {K_{i} S_{i} \left( \lambda \right)} $$

As in Eq. (1), $L\left( \lambda \right)$ represents the synthetic spectrum $S_{i} \left( \lambda \right)$ is involved in the spectral simulation unit spectrum. $K_{i}$ is the energy coefficient of the corresponding unit spectrum, that is, by adjusting the value of $K_{i}$, to achieve control of the unit spectrum radiation energy. As can be seen from Eq. (1), when the number of units involved in the simulation of the number of spectral bands, the better the monochromaticity, the greater the range of energy coefficient adjustment, the higher the spectral simulation accuracy and modulation capacity, the greater the radiation dynamic range. However, for a spectral imaging system, the higher the number of unit spectral bands and the better the monochromaticity, the better the imaging quality is required and the higher the complexity of the system. Therefore, beginning from the unit spectrum, we simulate and analyze the influence of the number of the unit spectrum and monochromaticity on the accuracy of the spectral simulation by combining Eq. (1) so as to provide theoretical guidance and ideas for system design.

By simulating the unitary spectrum segment $S_{i} \left( \lambda \right)$ using the Gaussian distribution model, $S_{i} \left( \lambda \right)$ can be expressed as:

2 $$ S_{i} \left( {\lambda_{i} } \right) = \tau \cdot \exp \left[ {\frac{{ - \left( {\lambda - \lambda_{i} } \right)^{2} }}{{2\omega^{2} }}} \right] $$

As in Eq. (2), where $\lambda_{i}$ is the peak wavelength, $\tau$ is the scale factor, and $\omega$ is the spectral half-peak width factor.

The number of spectral cell bands can be expressed in terms of spectral peak intervals. The denser the peak interval of the unit spectral bands involved in the simulation, the greater the number of them, and vice versa. The half-peak width of the spectrum can express the monochromaticity of the unit spectrum, and the better the monochromaticity of the unit spectrum involved in the simulation, the smaller the half-peak width. Different peak intervals and half-peak widths are simulated and analyzed by setting different values of $\lambda_{i}$ and ω to explore how they affect the accuracy of the spectral simulation. Three spectral half-peak widths of $\omega$ = 10 nm, 20 nm, and 50 nm, respectively, and three different characteristic parameters of half-peak widths of $\Delta \lambda$ = 5 nm, 10 nm, and 20 nm were used to simulate the spectral simulation accuracy of the 2700 K color temperature curve in the spectral range of 500–800 nm. The analysis results are shown in Table 1.

**Table 1:** Results of spectral simulation accuracy affected by the characteristics of the unit spectrum.

Interval half-peak width	$\omega$ = 10 nm (%)	$\omega$ = 20 nm (%)	$\omega$ = 50 nm (%)
Interval $\Delta \lambda$ = 5 nm	0.21	0.53	0.62
Interval $\Delta \lambda$ = 10 nm	0.23	0.53	0.69
Interval $\Delta \lambda$ = 20 nm	1.85	0.65	0.71

**Figure 2:**
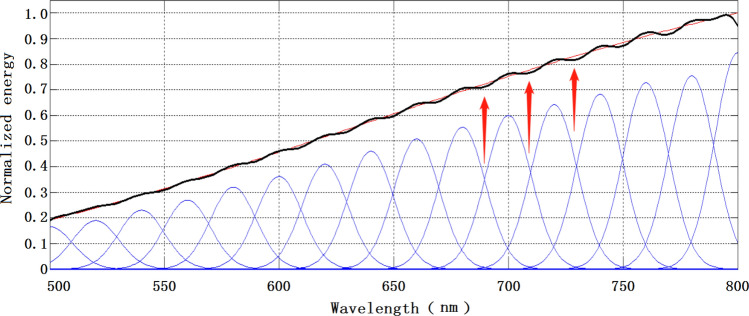
Half-peak width of 10 nm, 20 nm interval unit spectral simulation results (The red line is the ideal 2700 K color temperature curve, the blue line is the unit spectrum segment, and the black line is the 2700 K simulation curve).

As shown in Table 1, the simulation results of spectral simulation accuracy show that the first set of simulation results: when the spectral half-peak width of the unit spectral segment for simulation is ω = 10 nm, the spectral simulation accuracy is 0.21% when the peak interval $\Delta \lambda$ = 5 nm, 0.23% when the peak interval $\Delta \lambda$ = 10 nm, and 1.85% when the peak interval $\Delta \lambda$ = 20 nm; The second set of simulation results: when the spectral half-peak width of the unit spectral band for which the simulation is performed is ω = 20 nm, the spectral simulation accuracy is 0.53% when the peak interval $\Delta \lambda$ = 5 nm, 0.53% when the peak interval $\Delta \lambda$ = 10 nm, and 0.65% when the peak interval $\Delta \lambda$ = 20 nm; The third set of simulation results: when the spectral half-peak width of the unit spectral segment for which the simulation is performed is ω = 50 nm, the spectral simulation accuracy is 0.62% when the peak interval $\Delta \lambda$ = 5 nm, 0.69% when the peak interval $\Delta \lambda$ = 10 nm, and 0.71% when the peak interval $\Delta \lambda$ = 20 nm; It can be seen that no matter the first, second or third group, the simulation results show that the smaller the peak interval of the unit spectral band, the higher the simulation accuracy. And from the first two simulation results of the first, second, and third groups, it can be seen that the spectral simulation accuracy becomes lower as the peak interval and spectral half-peak width become larger. However, the third simulation of the first, second, and third groups show different results, i.e., under the premise of the same peak spacing (i.e., $\Delta \lambda$ are 20 nm), the simulation accuracy with half-peak width ω = 10 nm is lower than that with half-peak width ω = 20 nm and 50 nm, which is due to that the spectral width of the unit spectral segment cannot cover the entire adjusted spectral segment, that is, the spectra in the middle of the two-unit spectral segments cannot realize the effective modulation, as shown in Fig. 2.

It is clear from the analysis that the peak interval of the unit spectral band dominates the color temperature simulation accuracy when simulating the stellar color temperature. Due to the control selection and resolution limitation of DMD, it is also theoretically impossible to obtain an infinite number (that is, infinitely small spectral interval) of unitary spectral bands when performing spectral modulation. A better spectral simulation accuracy can be obtained when the spectral half-peak width > peak interval.

### Spatial division and dynamic range analysis of digital micromirror array

In order to have the accurate simulation of a large dynamic range stellar magnitude, the spatial light modulation device DMD needs to be partitioned. In this paper, the DLP6500 model DMD is selected as the spectral modulation device. Compared with other models of DMD, the DLP6500 has the advantages of high resolution and a large array surface, and its main parameters are shown in Table 2.

**Table 2:** DLP6500 model DMD main parameters.

Parameters	Numerical value
Resolution	1920 × 1080
Single pixel size	7.56 μm
Diagonal size	0.65inch

In partitioning the DMD spatial region, the larger the size of spatial region I, the more pixels it occupies, the higher its spectral resolution, and the higher the system spectral simulation accuracy. The larger the size of spatial region II, the more pixels it occupies, the larger the modulable multiplier, and the wider the dynamic range of the system. Given that the Large dynamic radiation spectroscopy system will produce residual aberrations during the design and mounting process, leading to problems such as spectral superposition and spectral line bending, and the DMD diffraction effect and the diffraction efficiency of the grating in the system will lead to the emergence of local peaks and valleys in the spectrum, first of all, we try to provide a large spatial area for the grating level 0 diffraction under the premise of ensuring the accuracy of the system spectral simulation. The DMD spatial region division ratio is 3:1. That is, the DMD spatial region is divided into four equal parts, with spatial region I accounting for 3/4 and spatial region II accounting for 1/4. At this time, the resolution of the system space region II is 480 × 1080, and the output energy is modulated by controlling the switching state of each micromirror, so the dynamic range of energy modulation in space region II is theoretically up to 5.184 × 105 (480 × 1080 = 518,400) times.

### Optical system design

#### Design ideas

**Figure 3:**
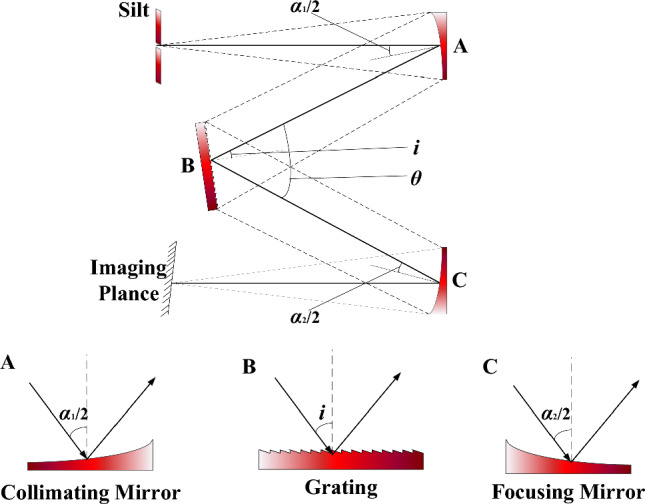
Schematic diagram of non-crossover Chenier Turner type optical structure.

The large dynamic range radiation spectroscopy system is divided into two parts, which are the spectral modulation optical system responsible for the accurate simulation of stellar spectra and the energy modulation optical system responsible for the large dynamic range adjustment of stellar stars, where the spectral modulation part and the energy modulation part share the grating, focusing mirror and DMD of the system. Spectral modulation system using imaging mirrors to sequentially align the spectra in the spatial region I of the DMD. The energy modulation system uses a planar reflector to image the beam in the spatial region II of the DMD. Spectral modulation systems are imaging systems, that have certain requirements for imaging quality. The energy modulation system is a non-imaging system and its imaging quality does not need to be considered. In order to meet the design requirements of the system, a non-crossing Chenier Turner-type structure was selected as the optical structure of the spectral modulation system. The Chenier Turner optical structure has more optimization variables to achieve better imaging quality. It has a certain space to accommodate the energy-modulated optical system reflector structurally compared to the crossed Chenier Turner type structure. The non-crossover Chenier Turner-type optical structure is shown in Fig. 3.

The aberrations that affect the imaging quality of non-crossed Czerny-Turner-type structures are spherical aberration, coma, and astigmatism. Among them, spherical aberration and coma affect the spectral imaging quality in the spectral dispersion direction, and astigmatism elongates the spectral spot in the non-dispersion direction. As the large dynamic range stellar analog optical system arranges the spectra of each spectral band sequentially on the DMD spatial array surface, there is no need to consider the effect of astigmatism on the spot imaging quality.

The spherical aberration will make the spectral spot wider, which can be compensated by adjusting the curvature of the focusing mirror. Coma will affect the spectral spot shape. When the coma is too large, it will also produce false spectral lines, bringing spectral line overlap and difficulties for spectral modulation. The non-cross Czerny–Turner type structure eliminates the coma equation, as shown in Eq. (3).

3 $$ \left\{ \begin{subarray}{l} \varepsilon_{1} = \left( {{{r_{2} } \mathord{\left/ {\vphantom {{r_{2} } 2}} \right. \kern-0pt} 2}} \right)\cos \left( {\alpha_{2} } \right)\delta \theta = \frac{{3W^{2} r_{2} \sin \left( {\alpha_{1} } \right)\cos \left( {\alpha_{2} } \right)\cos^{3} i}}{{8r_{1}^{2} \cos^{3} \left( {\alpha_{1} } \right)\cos \theta }} \\ \varepsilon_{2} = \left( {{{r_{2} } \mathord{\left/ {\vphantom {{r_{2} } 2}} \right. \kern-0pt} 2}} \right)\cos \left( {\alpha_{2} } \right)\left( {\frac{\partial F}{{\partial w^{\prime}}}} \right) = \frac{{3W^{2} \cos^{2} \theta \sin \left( {\alpha_{2} } \right)}}{{8r_{2} \cos^{2} \left( {\alpha_{2} } \right)}} \\ \varepsilon_{1} + \varepsilon_{2} = 0 \end{subarray} \right. $$

As in Eq. (3), where $\varepsilon_{1}$ is the coma width produced by the collimating mirror, $\varepsilon_{2}$ is the coma width produced by the focusing mirror, $\alpha_{1}$ is the off-axis angle of the main ray on the collimating mirror, $\alpha_{2}$ is the off-axis angle of the main ray on the focusing mirror, $r_{1}$_,_ and $r_{2}$ are the radii of curvature of the focusing and imaging mirrors, $i$ is the angle of incidence of the main ray on the grating, and $\theta$ is the diffraction angle of the central wavelength. $W$ is the grating width, $F$ is the optical range function.

Thus, the non-crossing Czerny-Turner type structure elimination coma condition can be derived:

4 $$ \frac{{\sin \alpha_{2} }}{{\sin \alpha_{1} }} = \frac{{r_{2}^{2} }}{{r_{1}^{2} }}\left( {\frac{\cos i}{{\cos \theta }}} \right)^{3} \left( {\frac{{\cos \alpha_{2} }}{{\cos \alpha_{1} }}} \right)^{3} $$

For the system coma, we can first determine the $\alpha_{1}$, $r_{1}$, $r_{2}$, $i$*,* and $\theta$ of the system and then balance the coma at each spectrum of the system by optimizing the off-axis angle $\alpha_{2}$ of the focusing reflector to achieve the correction of the coma.

#### Optics system optimization design

**Figure 4:**
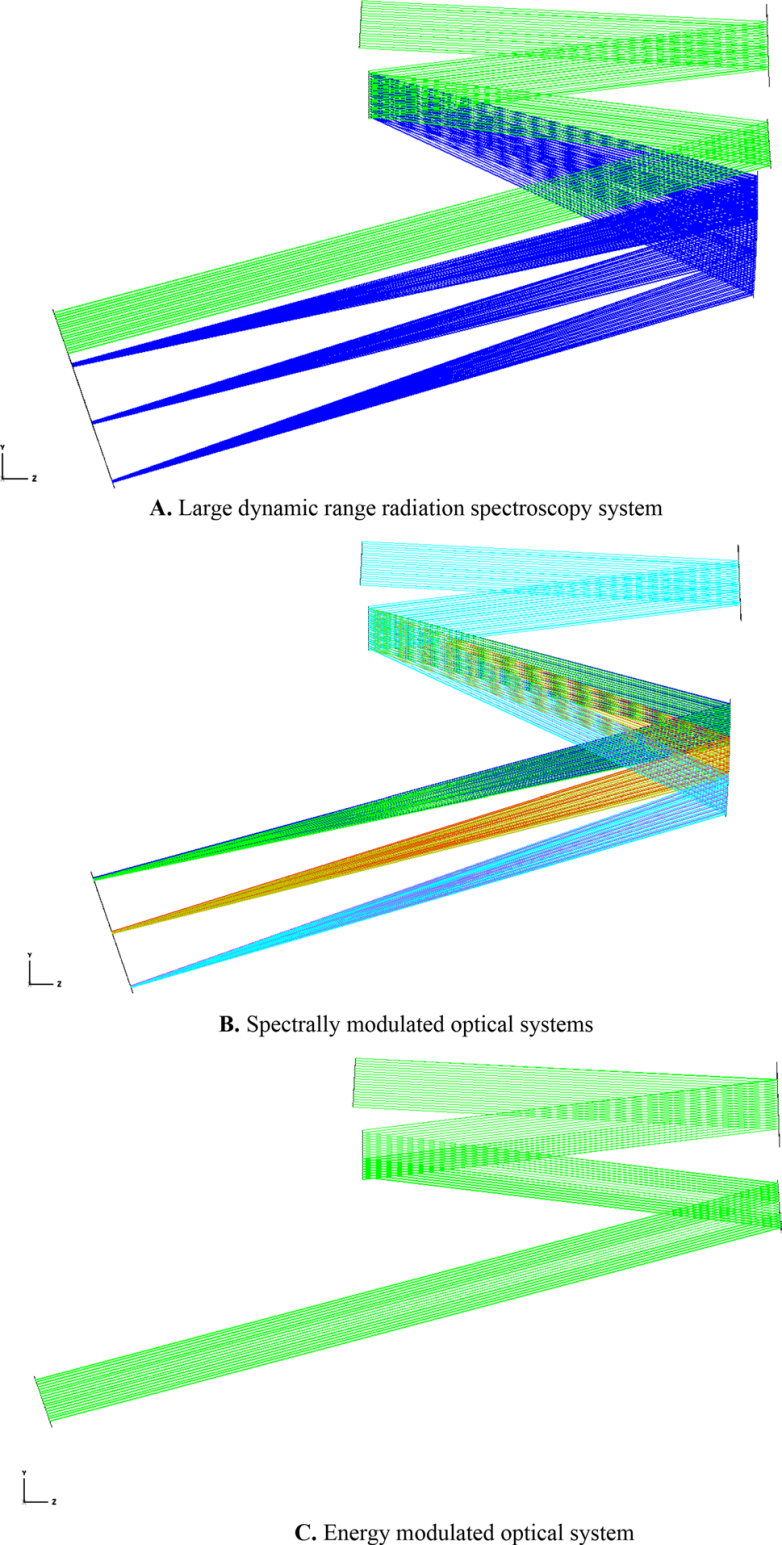
Large dynamic range radiation spectroscopy system optical path simulation schematic.

**Figure 5:**
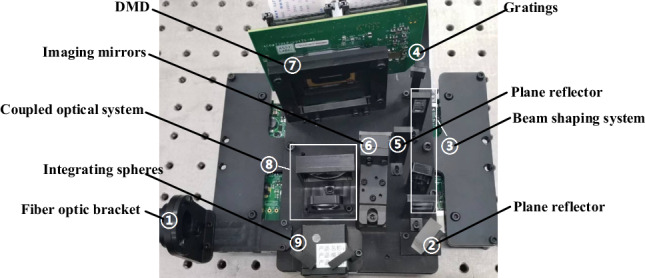
Physical image of large dynamic range radiation spectrometer system (①) depicts the incident fiber holder, which is crucial for securing the super-continuum laser's head. The planar reflector (②) folds the incident beam by 90°, aiding in size reduction and directing it into the beam shaping system (③). This system transforms a circular spot into a square one, ensuring full coverage of the DMD's column pixels for subsequent beam modulation. Components ①, ②, and ③ form the input light source system, corresponding to the light source system illustrated in Fig. 1. The gratings (④) separates the incoming mixed beam, allocating distinct spectral angles of emission. Both the 0th and 1st order diffraction beams from the gratings, are utilized. The plane reflector (⑤) intercepts and reflects the 0th order diffraction beam from the gratings to the DMD's edge area, forming a parallel square spot without dispersion. The Imaging mirror (⑥) captures the 1st order diffracted beam, sequentially projecting each spectrum onto the DMD as elongated, narrow-band light spots. The DMD (⑦) modulates both the 0th order diffracted square spot and individual spectral imageries, enabling large dynamic energy modulation and precise stellar spectrum simulation. Parts ④, ⑤, ⑥, and ⑦ constitute the large dynamic range radiation beam splitting system. The coupling optical system (⑧) integrates the DMD's outgoing beam into the integrating sphere (⑨). This sphere homogenizes the incoming beam, ensuring a uniform output.)

In this system, the laser collimated beam expander is used instead of the collimated objective lens in the non-crossed Chenier Turner type structure, and the system is optimally designed according to the comet aberration elimination condition, with the imaging mirror as the main optimization parameter to balance the comet aberration of the system. The design was completed using the software Zemax. The completed design of the large dynamic range radiation spectroscopy system simulation diagram is shown in Fig. 4, and the physical diagram is shown in Fig. 5. The optical path of the spectral modulation system is shown in Fig. 4B, and the optical path of the energy modulation system is shown in Fig. 4C. For the whole spectrum to be imaged both in the dispersion direction and tiled on the DMD array surface, the imaging mirror of the system adopts the form of a columnar mirror. That is, the light is focused and imaged in the dispersion direction, and only the light propagation direction is changed in the non-dispersion direction.

**Figure 6:**
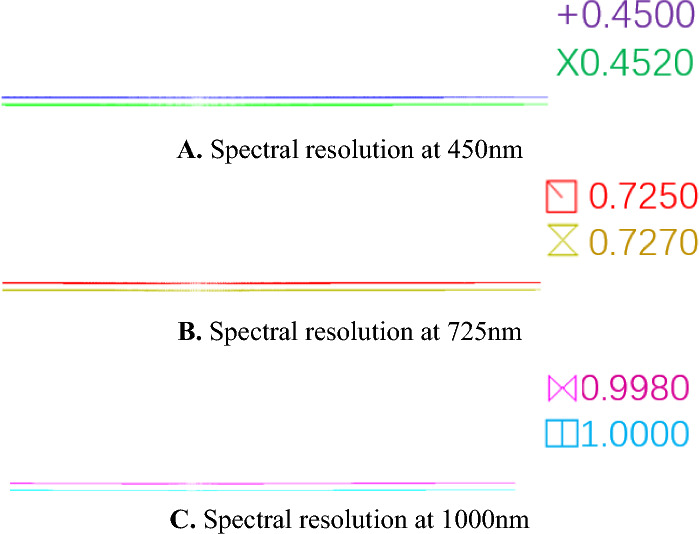
Spectral imaging system point column diagram.

**Figure 7:**
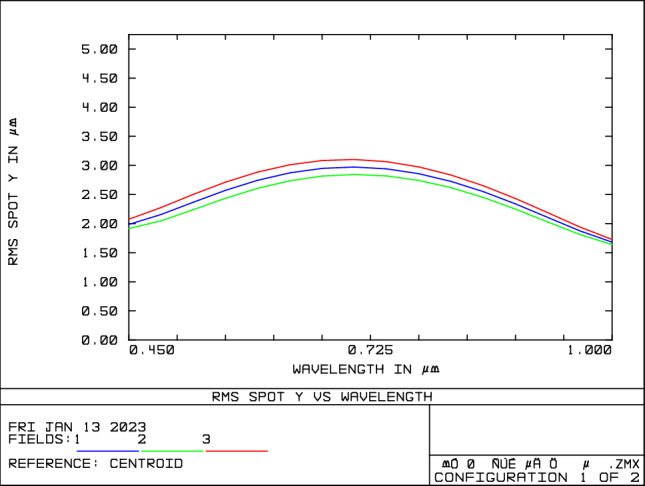
Spectral imaging optical system RMS versus wavelength curve along the dispersion direction.

Energy-modulated optical systems are non-imaging systems; therefore, only spectrally modulated optical systems are analyzed for image quality. The imaging quality of the spectral imaging system is shown in Figs. 6 and 7. Figure 6 represents the imaging position of the output spectra of the system on the image plane, i.e., for different spectra. According to Fig. 6, it can be seen that the system can separate 450 nm and 452 nm, 725 nm and 727 nm, 998 nm and 100 nm, and the spectral resolution of the system is 2 nm. The imaging of individual spectra is straight and without bending, and the system can well separate 450 nm and 452 nm, 725 nm and 727 nm, 998 nm and 100 nm, which means that the spherical aberration and coma correction is good. It can be seen from Fig. 7 that the spot size along the y-direction is less than 3.5 μm in the whole spectral range, and the imaging quality of the system is good.

The optical parameters of the completed design of a Large dynamic range radiation spectrooptical system are shown in Table 3.

**Table 3:** Parameters of optical system.

Serial number: face	Radius	Distance	Parameters		
			X-deflection angle	Grating constants	Diffraction level
1	∞	43 mm	–		
2	∞	42.25 mm	− 6		
3 (Ganting)	∞	42.25 mm	7°	300 lp/mm	− 1
4					
Structure 1	− 159 mm	70.7 mm	− 5		
Structure 2	∞	–	5°		
5 (DMD)	∞	–	− 16		

## Experimental test and results

**Figure 8:**
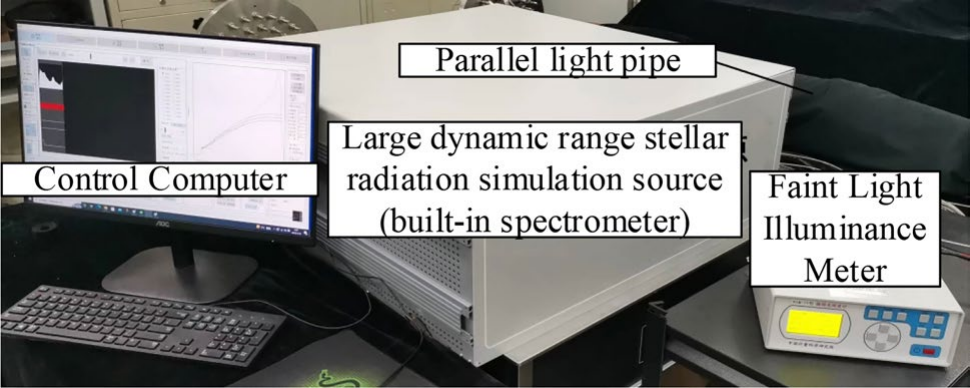
Large dynamic range stellar radiation simulation testbed.

The large dynamic range stellar radiation simulation testbed is built as in Fig. 8. The test platform mainly consists of a light source system (Built into a large dynamic range radiation spectroscopy system), a Large dynamic range radiation spectroscopy system, a Parallel light pipe, a Spectrometer (Built into a large dynamic range radiation spectroscopy system), and low light illuminance meter composition. Among them, the purpose of the parallel light tube is to output the infinity star simulation beam, the spectrometer is to test the color temperature simulation accuracy, the faint illuminance meter is used to test the star magnitude simulation accuracy, and the large dynamic range test of the system relies on the faint illuminance meter to calibrate the bright stars and cooperate with the spectrometer for the indirect measurement of the faint stars.

The large dynamic range stellar radiation simulation test platform is shown in Fig. 8. The test platform mainly consists of a light source system (built inside the large dynamic range radiation spectroscopy system), a large dynamic range radiation spectroscopy system, parallel light tubes, a spectrometer (built inside the large dynamic range radiation spectroscopy system), and a faint light illuminometer. In particular, the purpose of the parallel light pipe is to output an infinity star simulation beam, the spectrometer is to test the color temperature simulation accuracy, the faint light illuminometer is used to test the magnitude simulation accuracy, and the large dynamic range test of the system relies on the faint light illuminometer to calibrate the bright stars, together with the spectrometer for the indirect measurement of the faint stars.

The main parameters of each part of the built system are shown in Table 4.

**Table 4:** Main parameters of each part of the build system.

Ultra-continuum laser source main parameters	
Total power	> 8 W
Wavelength range	430–2400 nm
Pulse energy	> 1 µJ
Visible power	> 1000 mW
Power Stability	< 1%
spot dispersion angle	< 1 mrad
DMD main parameters	
Model number	DLP6500
Resolution	1920 × 1080
Single pixel size	7.56 µm
Diagonal dimension	0.65inch
Micromirror declination	± 12°
Spectrometer main parameters	
Model number	AvaSolar 1
Spectral range	300–1100 nm
Spectral sampling interval	0.6 nm
Spectral accuracy	± 0.2 nm
Parallel light tube main parameters	
Monitoring range	1000 mm
Field of view	0.5°
Caliber	100 mm
Spectral range	400–1100 nm
Faint light illuminance meter main parameters	
Dynamic range	1 × 10^–8^–2 × 10^0^* lx*
Uncertainty/accuracy class	Urel = 1.6%, (k = 2)

### Color temperature simulation accuracy test

The color temperature simulation mainly relies on the spectral modulation system in the large dynamic range stellar radiation simulation testbed to complete the test simulation process as follows:

1. Calibration of each column in the DMD spatial region I corresponds to the cell spectrum, and for the completed DMD, its column pixels represent the cell spectrum $S_{i} \left( \lambda \right)$.The row pixels represent the weight coefficients $K_{i}$.
2. Solve for the weighting coefficients, compare the full spectrum with the target color temperature spectrum, and calculate the weighting coefficients $K_{i}$ at different wavelengths.
3. Control the switching states of the corresponding row pixels at different wavelengths (i.e., for different column pixels of the DMD) according to the weighting coefficients to realize the simulation of the target spectrum.
4. Comparison of the simulated and ideal color temperature curves using a spectrometer.
5. Repeat the steps 2–4 for the fine adjustment of the local modulation overrun part until the simulated target spectral curve meets the usage requirements.

**Figure 9:**
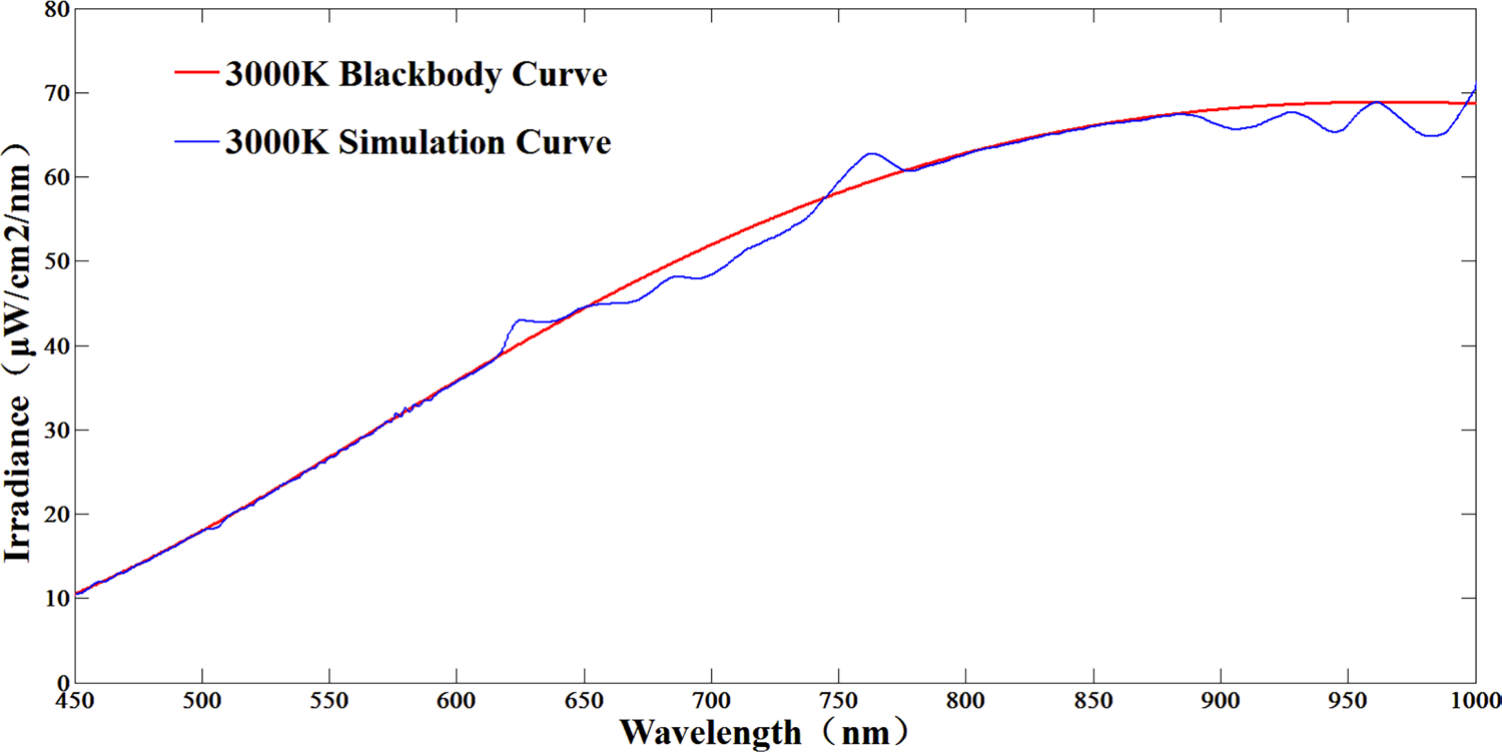
3000 K color temperature simulation results.

**Figure 10:**
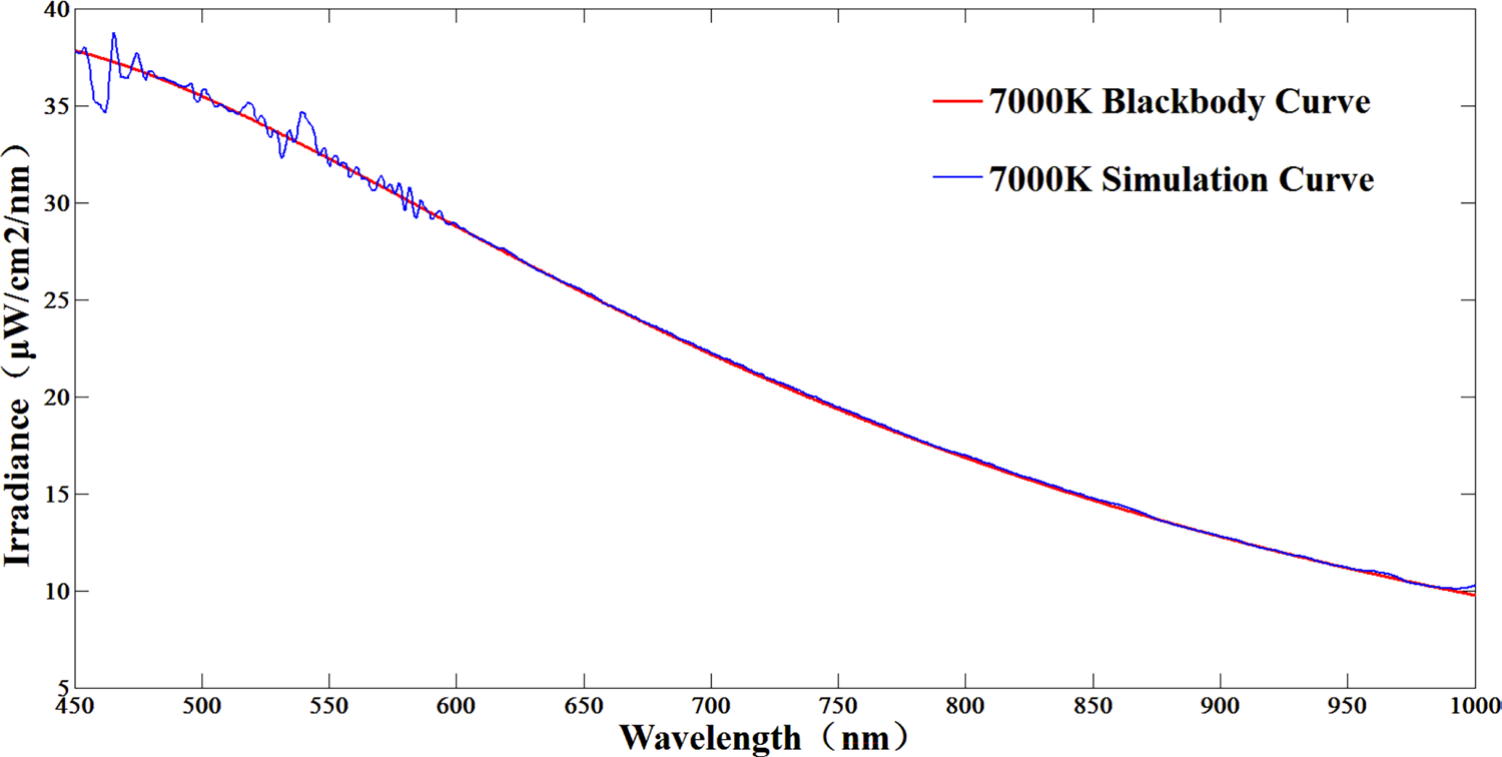
7000 K color temperature simulation results.

**Figure 11:**
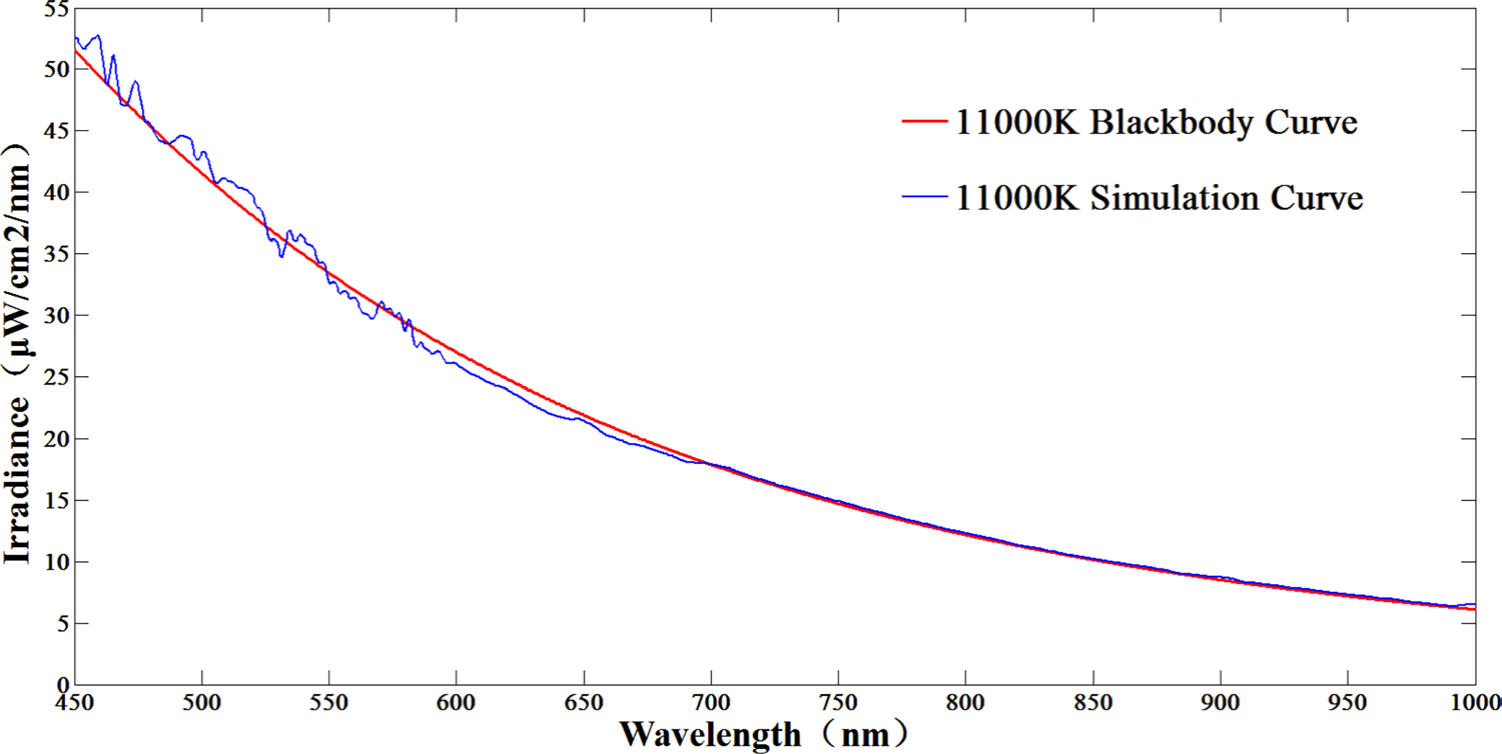
11,000 K color temperature simulation results.

In the color temperature range of 3000–11,000 K. Three typical color temperatures of 3000 K, 7000 K and 11,000 K were selected for simulation. The simulation results are shown in Figs. 9, 10 and 11. The red line is the simulated spectral curve, and the blue line is the theoretical color temperature curve.

According to Figs. 9, 10 and 11, the maximum simulation error of the system at 3000 K color temperature is − 6.9%, which occurs at 700 nm; the maximum simulation error of the system at 7000 K color temperature is − 6.95%, which occurs at 460 nm; the maximum simulation error of the system at 11,000 K color temperature is 6.82%, which occurs at 455 nm; thus, it can be seen that in the color temperature range of 3000–11,000 K, the simulation error of the system is better than ± 7%. 11,000 K range, the system color temperature simulation error is better than ± 7%.

### Magnitude simulation range and accuracy test

Simulation of the system magnitude is tested. Magnitude simulation is performed under the premise of ensuring the accuracy of color temperature simulation. Therefore magnitude simulation requires the interplay of a spectral modulation system and a large dynamic range magnitude modulation system. The system magnitude is measured directly using a low light illuminance meter, which has a measurement limit of 1.0 × 10^−8^ *lx*, so a direct measurement of 0 to + 6 Mv can be achieved. For + 6 to + 12 Mv, the indirect measurement method of the spectrometer is used. The test steps are as follows:

1. Take 7000 K color temperature as an example. Its irradiance is the area enclosed by the spectral curve and the coordinate axis. Different magnitudes have different irradiance, and the color temperature curve under the same magnitude is also unique, so a one-to-one correspondence between the magnitude value and the area enclosed by the spectral curve and the coordinate axis can be established.
2. The spectral curve of 0Mv at 7000 K color temperature is used as the standard, The color temperature curve is quantitatively (2.512 times or a multiple of 2.512) attenuated based on the 2.512 times difference in illumination of adjacent stars, The corresponding area is also attenuated by the same multiple, so that the theoretical color temperature simulation curve of + 7 to + 12 Mv can be obtained.
3. The theoretical color temperature simulation curve of + 7 to + 12 Mv is targeted, and the spectral modulation system and energy modulation system are used to simulate the theoretical color temperature curve of + 7 to + 12 Mv with each other.
4. The completed curve of the simulation is solved by area integration, and then compared with the theoretical area to derive the corresponding simulated star equivalents.

**Figure 12:**
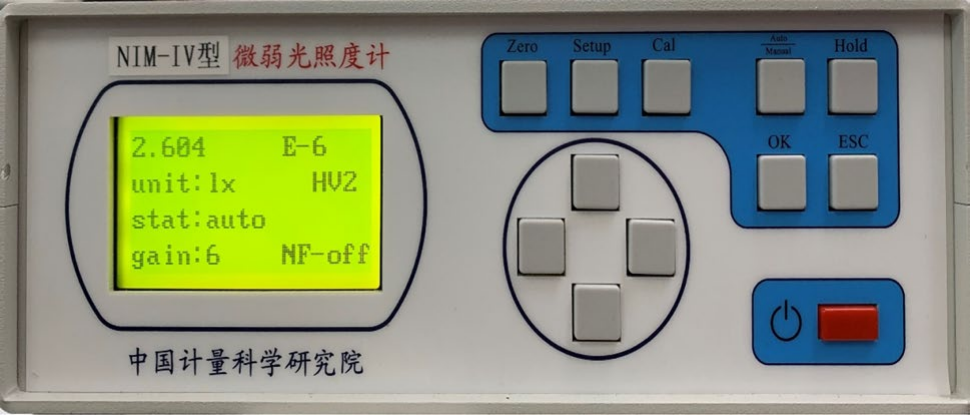
0 Mv simulation results.

**Figure 13:**
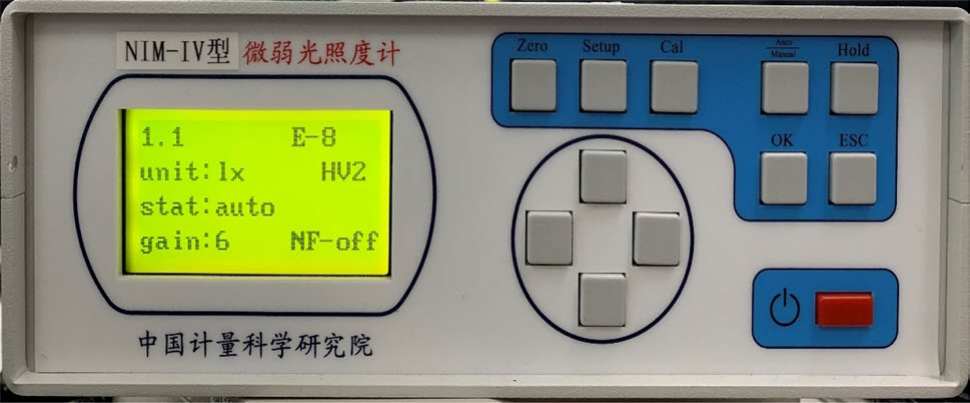
+ 6 Mv simulation results.

The 0 Mv and + 6 Mv of the simulated 7000 K color temperature were measured using a low-light illuminometer. The 0Mv measurement was 2.604 × 10^-6^* lx*, and the + 6Mv measurement was 1.1 × 10^−8^* lx*, and based on the data, it can be calculated that the magnitude simulation errors were + 0.028 Mv and + 0.038 Mv, respectively. The magnitude measurements are shown in Figs. 12 and 13.

**Figure 14:**
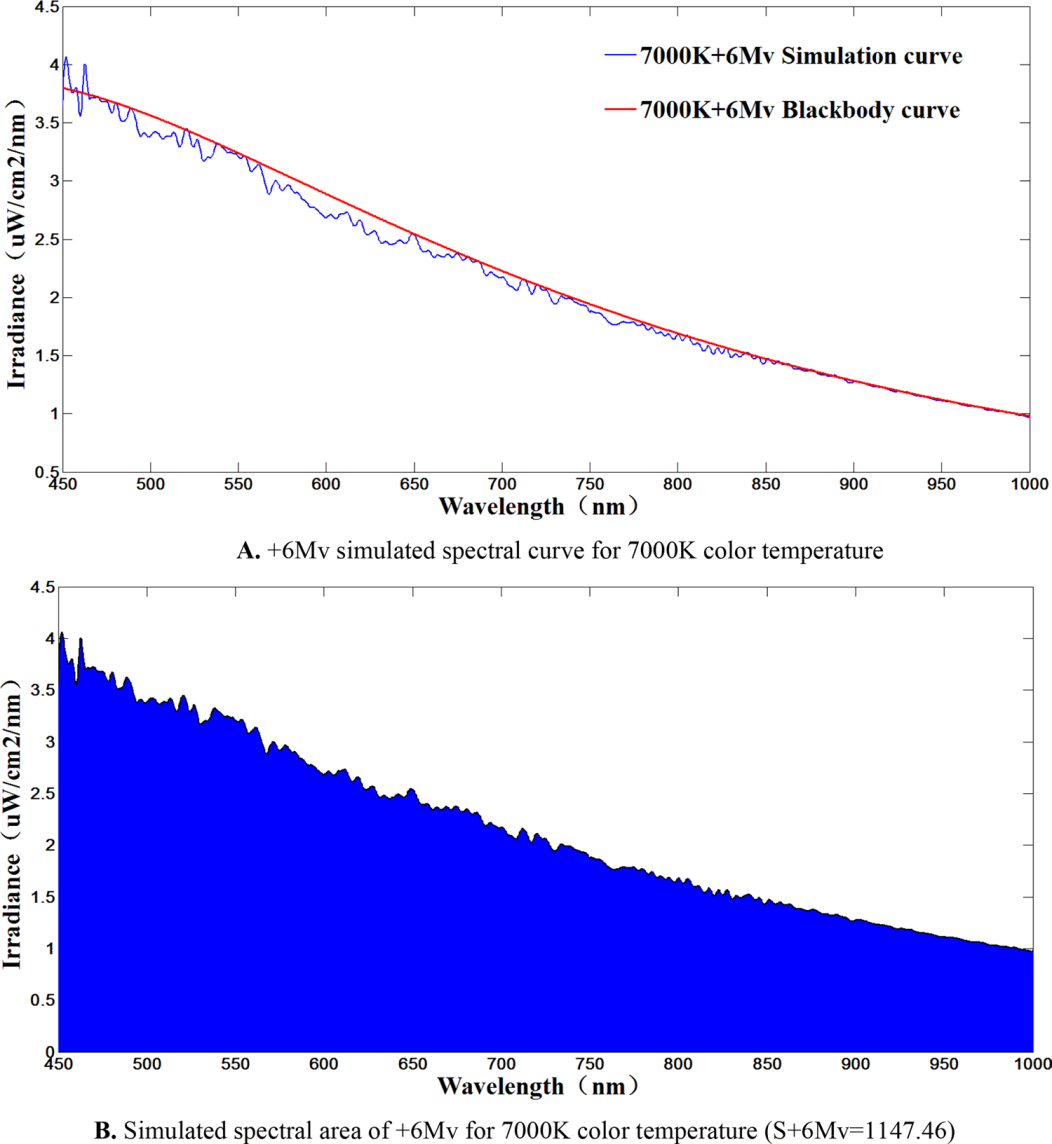
+ 6 Mv color temperature test curve at 7000 K.

**Figure 15:**
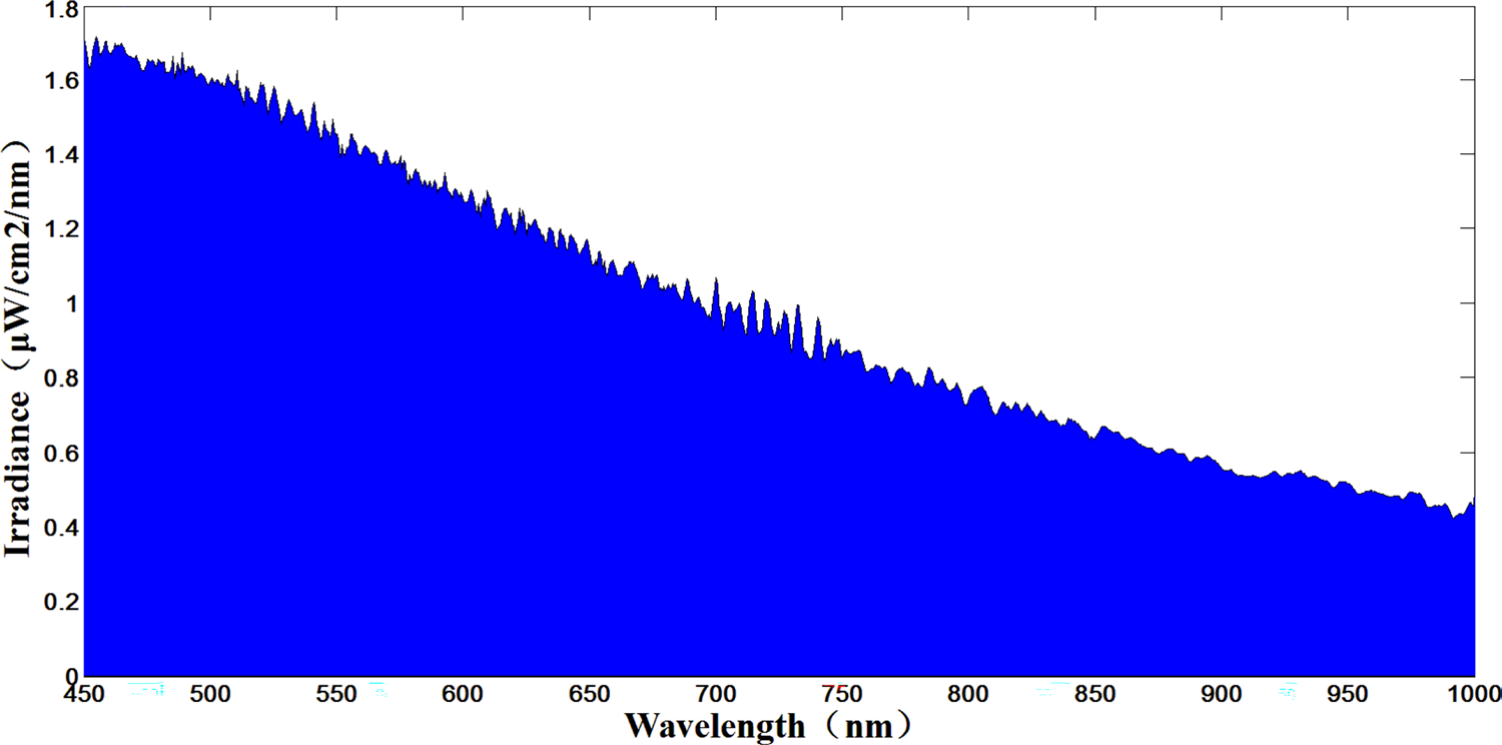
+ 7 Mv test curve for 7000 K color temperature (S_+ 7 Mv_ = 470.95).

**Figure 16:**
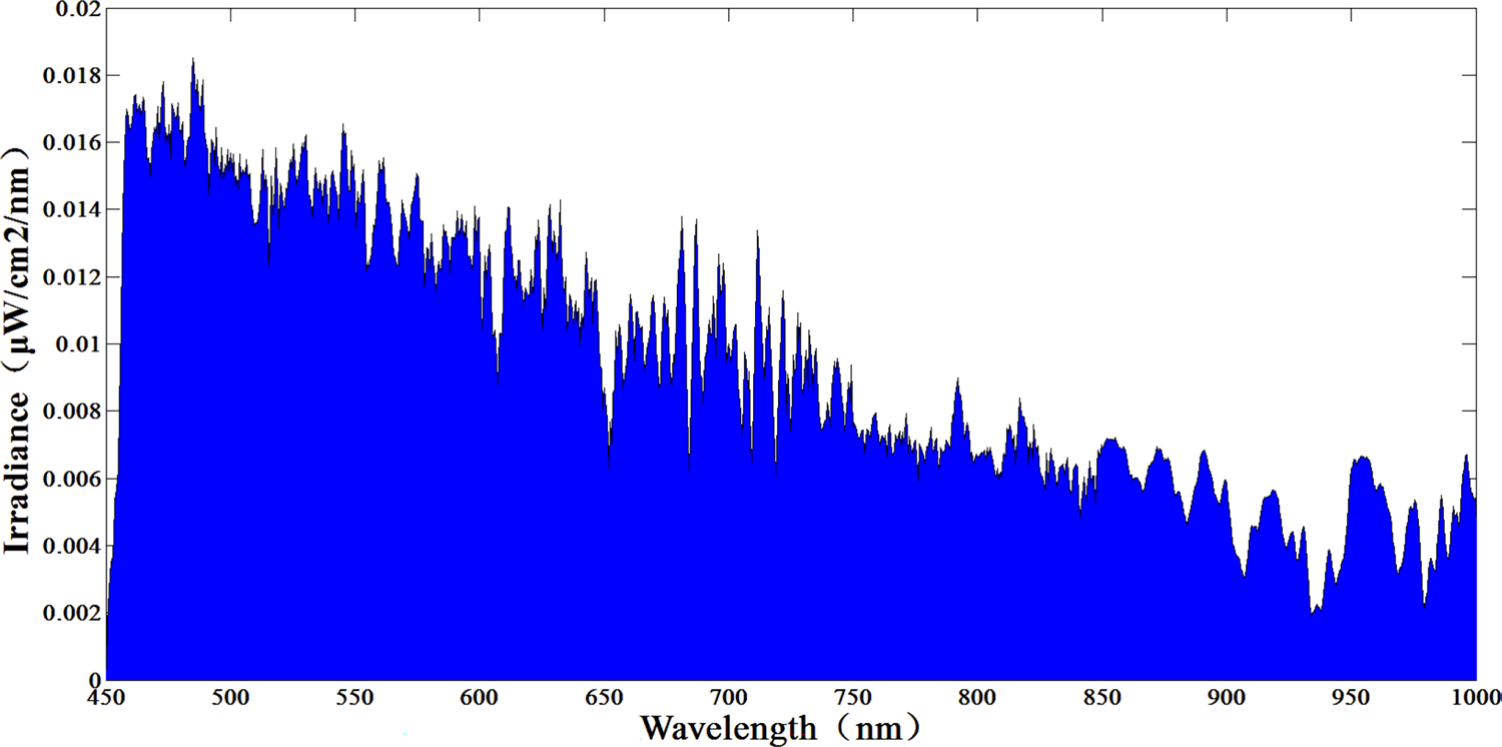
+ 12 Mv test curve for 7000 K color temperature (S_+ 12 Mv_ = 4.35).

Indirect measurement of + 7 Mv and + 12 Mv using the + 6 Mv spectral curve at 7000 K color temperature as a reference. The test results are shown in Figs. 14, 15 and 16. Where Fig. 14 shows the + 6 Mv spectral curve for 7000 K color temperature. The spectral curves of + 7 Mv and + 12 Mv are shown in Figs. 15 and 16, respectively. The area integral and area ratio show errors of + 0.03 Mv and − 0.047 Mv for the starlight level simulations at + 7 Mv and + 12 Mv. respectively. According to the test results, the system can realize the accurate simulation of large dynamic range stars from 0 to + 12 Mv, i.e., the dynamic range of irradiance values can be changed 63,130 times.

### Narrowband spectral output test

**Figure 17:**
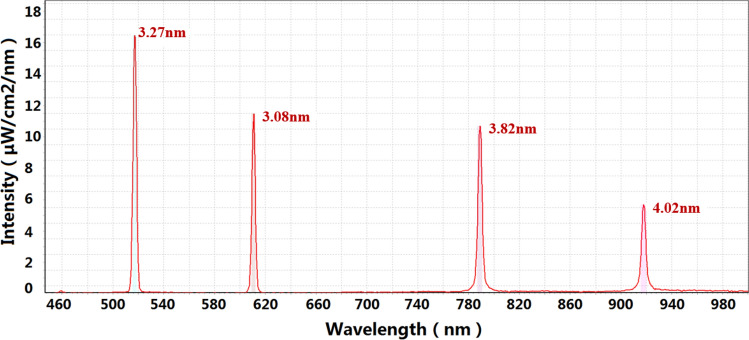
Narrowband mode test results.

The system was tested in narrowband mode, with a single column of pixels as a group, and 4 groups of pixels on the face of the DMD array were randomly selected for testing. The test results are shown in Fig. 17.

The spectral data of the narrow-band mode are shown in Table 5. According to the data in Table 5, the maximum half-peak width of the output spectrum in the narrow-band mode is 4.02 nm. It can be seen that the system has good monochromaticity of narrowband output beam and can be used for monochromatic beam simulation.

**Table 5:** Narrowband mode test data.

Parameter	Data			
Wavelength (nm)	517.07	601.83	789.14	917.85
FWHM (nm)	3.27	3.08	3.82	4.02

## Conclusion

This article proposes a design method for a large dynamic range stellar radiation simulation optical system, which combines wide spectral high-resolution subdivision and spatial light partition modulation to address the low spectral simulation accuracy, small energy adjustment range, and single function issues in existing stellar spectral simulation technologies. This method realizes accurate simulation of wide-band stellar spectra, large dynamic range adjustment of star magnitudes, and narrow-band monochromatic beam output in a single system, expanding the system's working mode and application range. A large dynamic range radiation spectrophotometric optical system is designed, which achieves a Y-direction RMS radius of less than 3.5 μm and spectral resolution better than 2 nm through imaging analysis, thus realizing an accurate subdivision of wide-band light beams. With the spatial light modulation characteristics of the DMD, the subdivided beams are accurately modulated to achieve the purpose of simulating the target spectrum. An experimental platform is constructed to test the system. The test results show that the system has a spectral range of 450–1000 nm and a color temperature simulation range of 3000–11,000 K. The color temperature simulation error is better than ± 7%. With the accuracy guarantee, the star magnitude simulation range is 0 to + 12 Mv (a change of 63,130 times in irradiance), and the star magnitude simulation accuracy is better than ± 0.05Mv. The half-width of the narrow-band mode output beam is better than 4 nm. The system can realize the accurate simulation of stellar color temperature, large range of energy adjustment, and accurate output of narrow-band monochromatic beams, expanding its application range. It has engineering application value in the fields of aerospace^27^, spectral calibration^19^, radiation dosimetry^28^, colorimetry, light information, biochemistry, and other research fields.

The current system is capable of accurately simulating stellar radiation information. Still, when faced with spectral curves with complex spectral characteristics, the spectral modulation capability of the system has some limitations. This is due to the reduced number of physically modulatable pixels on the DMD array after partitioning, which to some extent compresses the imaging spectral surface of the spectral modulation system, leading to a decrease in spectral resolution and a decrease in modulation accuracy under the same system parameters, making the system inadequate for dealing with complex spectral distributions (such as solar spectra). In comparison with the traditional LED hybrid simulation system, although it has some advantages in dynamic range and spectral simulation accuracy, the output energy is weak, and high energy simulation cannot be realized. Therefore, future research will focus on reducing residual system aberrations and improving spectral resolution, thereby enhancing spectral modulation capability. And how to boost system energy.

## Data Availability

All data generated or analyzed during this study are included in this published article.

## References

[1] Cheng F, Zheng X (2008). Effect of spectral non-matching on the calibration accuracy of optical remote sensors. Opt. Precis. Eng..

[2] Xu D (2022). Design of a digital tunable stellar spectrum calibration source based on a digital micromirror device. Measurement.

[3] Liu J (2015). Star extraction based on clustering within a star tracker. Sci. Sin. Technol..

[4] Jiang Z, Han D, Yuan T, Xie F, Chen J (2004). Study on auto focusing algorithm for automatic microscope. J Image Graph.

[5] Wang D, Li M, Huang X (2022). Spacecraft observability theory based multi-source fusion autonomous navigation technology. Sci. Technol. Foresight.

[6] Zhang W (2020). A study of the navigation technology and application based on astronomical spectral velocity measurement. Navig Control.

[7] Rucinski S, Carroll K, Kuschnig R, Matthews J, Stibrany P (2003). Most (microvariability & oscillations of stars) Canadian astronomical micro-satellite. Adv. Space Res..

[8] Hackett, J. & Li, L. Sapphire-like payload for space situational awareness. In *Advanced Maui Optical and Space Surveillance Technologies Conference 2012 (AMOS 2012)*.

[9] Wallace, B. *et al*. The near earth object surveillance satellite (NEOSSat). In *Conference on Applications of Photonic Technology* 5578.

[10] Laurin, D., Hildebrand, A. R., Cardinal, R. D., Harvey, W. & Tafazoli, S. NEOSSat: A Canadian small space telescope for near Earth asteroid detection. In *Proceedings of SPIE* (2008).

[11] Simms, L. et al. Optical payload for the STARE pathfinder mission. In *SPIE Defense and Security *vols 8044: 804406 (2011).

[12] Zhang H, Zhou X, Wang X, Tian H (2020). Survey of technology status and development of all-time star sensors in near-earth space. Acta Aeronaut. Astronaut. Sin..

[13] Jianchao J (2023). Lightweight and high-sensitive optical camera technology for faint space target detection. Infrared Laser Eng..

[14] Zang X, Zhang J, Yang J, Zhang L, Zhang JL (2018). Research on multiple star magnitude simulation of star simulator. Laser Infrared.

[15] Da X, Shi-xin Y, Guo-yu Z, Gao-fei S, Jian Z (2020). Design of an Offner convex grating radiation calibration light source with a wide dynamic range. Chin. Opt..

[16] Li M, Han B, Liu H, Yuan L, Huang D (2020). Study on calibration technology of large field of view multi-star simulator. J. Changchun Univ. Sci. Technol..

[17] Fateley WG, Hammaker RM, Deverse RA (2000). Modulations used to transmit information in spectrometry and imaging. J. Mol. Struct..

[18] MacKinnon N, Stange U, Lane P, MacAulay C, Quatrevalet M (2005). Spectrally programmable light engine for in vitro or in vivo molecular imaging and spectroscopy. Appl. Opt..

[19] Brown SW, Rice JP, Neira JE, Johnson BC, Jackson JD (2006). Spectrally tunable sources for advanced radiometric applications. J. Res. Natl. Inst. Stand. Technol..

[20] Hongxing L (2012). Design of integrating sphere solar spectrum simulator based on xenon lamp and LEDs. Guangxue Jingmi Gongcheng (Opt. Precis. Eng.).

[21] Yinlin Y (2013). Design and test of a spectrally tunable integrating sphere reference light source with large exit aperture. Acta Opt. Sin..

[22] Jing-xu SUN (2015). 4 m extended uniform source for radiometric calibration. Chin. Opt..

[23] Hongxing L, Jianwei R, Zexun L, Zhi W, Baoyong Li (2015). LED-based single star simulator with multi-color-temperature and multi-star-magnitude output. Acta Opt. Sin..

[24] Luo, D. et al. Optical unmixing using programmable spectral source based on DMD. In *Conference on Next-Generation Spectroscopic Technologies* IX 98550P.1–98550P.9 (2020).

[25] Wang X, Li Z (2018). A spectrally tunable calibration source using Ebert–Fastie configuration. Meas. Sci. Technol..

[26] Zhu, J. *Study on Spectral Tunable Light Source Based on LED* (2010).

[27] Brown SW (2005). LED-based spectrally tunable source for radiometric, photometric, and colorimetric applications. Opt. Eng..

[28] Rice, J. E., Brown, S. S. & Neira, J. E. Development of hyperspectral image projectors (2006).

